# A novel research method to measure the usage of web-based information

**DOI:** 10.1016/j.mex.2022.101937

**Published:** 2022-11-24

**Authors:** Tariq S. Almontaser

**Affiliations:** a Faculty of Business and Law, Coventry University, Coventry, UK; b The College of Applied Administrative and Financial Sciences, Tripoli, Libya

**Keywords:** Web traffic, The usage of web-based information, Stakeholders’ engagement, Web metrics

## Abstract

This research method contributes to the literature by measuring commensurably ‘the usage of information for (or web traffic on) web-based research studies’. The introduced method deepens the understanding the functionality of online media by focusing on specific web metrics to make the usage of this type of media efficient for disclosure function. The inputs of new method are originally based on publicly available data, and it can be applied through 3 consecutive steps. Accordingly, this method is applicable to assess stakeholders’ engagement for any web-based research study. In sum, this method presents:

• The inputs of the method are publicly available data.
• This method is applicable for variety web-based research studies regardless of the applied methodological approach (e.g., qualitative, quantitative).

## Graphical abstract

**Figure:**
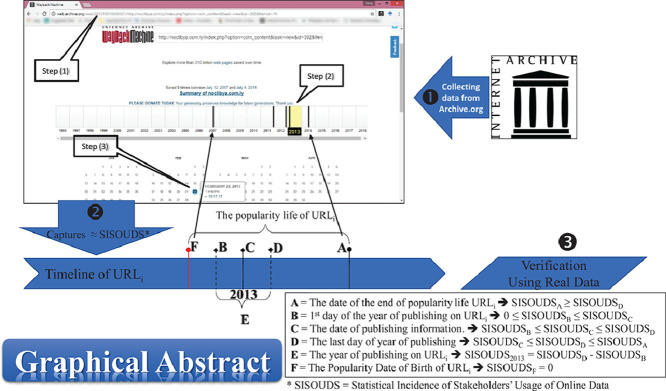

Specifications table

**Table:** 

Subject area:	
More specific subject area:	*All web-based research studies.*
Name of your method:	*The usage of web-based information.*
Name and reference of original method:	*The novelty of the method is based on adopting new source of data that has not been used before for this purpose. Accordingly, this method does not develop ‘original’ method/technique.*
Resource availability:	www.archive.org

## Introduction

This paper explores the assessment of web disclosure by determining and analyzing stakeholders’ usage of (or web traffic on) online CSR information (published on web 1.0 websites) as an indicator of their engagement. Web traffic is the amount of data received or sent by visitors, mostly stakeholders [4]. Their traffic [despite its supposed ambiguous identification] is the motivation of most websites’ reconstructions and improvements [9]. In this context, this paper has experienced two technical challenges: first, introducing an applicable tool/model to estimate the usage of (or web traffic on) information. Secondly, if applicable, identifying the probable influence of the ambiguity that might bias the outputs of the introduced method due to the *non*-*stakeholder* usage of CSR information. It is worth stating that the information of the *real* web traffic statistics is protected by many conventions, laws, and regulations that restrict personal data usage and information registration^1^. In other words, the *real* web metrics of websites are not publicly available.

### Literature review

The literature of *estimating* web traffic is based on retrieving data from popular directories, such as Alexa [14], Compete [17], or SimilarWeb [14]. In other words, many popular websites (web-directories) provide their users with such data. However, some provide it only for website owners (e.g., Google Analytics) or provide chargeable web traffic data for home pages only (e.g., Alexa, SimilarWeb), or make the data available only for global companies (e.g., Compete). Archive.org is the only public data source that freely serves^2^ its users with data *equivalents*, as I argue in this paper, to web traffic data related to millions of websites worldwide, including those of all the oil companies in the research sample. Archive.org has never been used, as shown in the research method, for this purpose. From a web metrics perspective, the extent of stakeholders’ engagement might be associated with time spent to navigate the website or specific web pages [6], the number of visits [8], the number of page views per visitor [2], the number of returning visitors [12], or the number of links on the website [18]. However, no research studies stakeholders’ engagement using real/estimated web traffic that this paper is uniquely seeking to estimate.

As mentioned before, the second technical challenge in this research study is identifying the probable influence of the analytic ambiguity, which might bias the introduced method's outputs due to the nested *non*-*stakeholder* usage of CSR information. The visitors of websites, in general, can be considered as firms’ *stakeholders* despite websites being public and free to access. The validity of this assumption is based on first prioritising the potential interests of targeted stakeholders on the reconstructed websites [and their web contents] [5]. Secondly, implementing web traffic by attracting *irrelevant* stakeholders, such as adding irrelevant content to increase website ranking via tagging, is an unsustainable tactic [7]. Accordingly, significant visitors, which are mostly firms’ stakeholders, are neither recognised nor attracted to the websites randomly. Non-relevant visitors (non-stakeholders) are unlikely to navigate beyond the home page, where most of the main gates of web hierarchy are located, and CSR information is limitedly published [10].

The website's popularity might lead to a significant change in the structure of relevant users [stakeholders] in terms of the usage of information. Web visitors from search engines, where websites likely occur in random searches, are two-thirds of the total of all visitors and have the longest session span compared to direct visitors [11]. Ortega and Aguillo (2010) found that search-engine visitors are more relevant to websites than direct visitors, presumably visiting websites on purpose. Accordingly, the irrelevant visitors [*non-stakeholders*] are mostly neither direct nor search-engine visitors who consist of mostly web visitors (Ortega & Aguillo, 2010; Pakkala et al., 2012). On the contrary, Plaza [13] found that both direct and search-engine visitors are, respectively, the most significant users [stakeholders] of web information from a visit-length perspective. Perhaps, this is because the studied website is related to scholars’ [local] community in Bilboa City^3^. In terms of penetration-type visitors, this example could be close to the firms chosen in the research sample of this paper in that they are domestic oil companies. Therefore, it can be observed that direct and search-engine visitors are generally most websites’ visitors (stakeholders). The identities of important stakeholders are based on whether, firstly, the *content* of websites targets global stakeholders. Consequently, search-engine visitors are the most significant stakeholders without dragging away the significance of direct-visiting stakeholders who obviously do not visit websites randomly. Secondly, if the *content* of websites targets domestic stakeholders, then both direct and search-engine visitors are the significant stakeholders.

### Detecting the traffic on web disclosure

This section demonstrates a unique contribution to the web-based literature in general. CSR-related information shall be the first application of measuring the stakeholders’ engagement by determining their *usage* of specific information using a publicly available resource of data. To determine the extent to which obtaining such information is difficult, Zotano et al. [20] developed their findings of ‘mass media websites’ using the web metrics of just one website of a popular TV channel in Spain. Moreover, this data is related to the main domain (home page) and does not reflect the web traffic on the other web pages branched from the home page. In general, most sources of real web traffic data that shall be used in the verification test in the next section cover a very limited time span for a few firms.

Obtaining detailed web traffic statistics of CSR information published on 13 bilingual websites—within eight years—owned by oil companies working in a context where many researchers have experienced serious data-collection difficulties [1] could be extremely challenging. Accordingly, a search for alternative (and applicably used) data sources of web traffic statistics is essentially needed. As illustrated before, Archive.org is used uniquely for this purpose. The official ‘terms of use’^4^ web page of Archive.org states in the third paragraph that ‘… *In using the Archive's site, Collections, and/or services, you further agree (a) not to violate anyone's rights of privacy*.’ Accordingly, the statistical ‘Collections’, the ‘number of captures’ (or snapshots) of the studied websites during the research time horizon, have been consistently used in this paper for academic purposes. Consequently, all analytic methods, techniques, and collected data of this paper are either introduced or utilised to generally contribute to the academic literature and support the studied context in particular. However, the ability to use the ‘number of captures’^5^ as an indicator of ‘web traffic’ has raised a challenging assumption: ‘**whether the number of captures accurately measures web traffic or web popularity**.’

According to Archive.org, their mechanism for capturing websites states, ‘*Internet Archive's crawls tend to find sites that are well linked from other sites’*^6^. This is called an ‘in-links’ approach to estimate site traffic created originally by the stakeholders themselves [3]. To confirm the case of data retrieved from Archive.org, analysing *real* website metrics could be the most appropriate approach to verify the associability between the numbers of captures (snapshots) of webpages, which have been presented on Archive.org during a period of time, and its *real* web traffic during the same period. I have obtained *real* web traffic data from four different sources, as shown in Table 1.

**Table 1:** The correlation between the number of Snapshot (captures) as found at Archive.org and the real data of web traffic on the website of the same firm during the same period.

Refs. (Source of Data)	Timeline of Data	The Name of the Firm (its Website)	Type of data	Correlation
([20], p. 52)	Three months; July 2014, February 2015 and February 2016	Radio Televisión Española RTVE (www.rtve.es)	[# of viewed] *Pages*	0.22***
			[No. of users’] *Visits*	0.76*
Almontaser Office	Seven years; from 2009 to 2015	Almontaser Office (www.almontaser.com)	Web traffic (in Giga Byte)	0.83***
([19], p. 150)	The aggregated data in daily basis (weekdays) from 1st of January to the 7th of July 2002.	Monach University (www.monash.edu)	[# of] *Requests from web users*	0.34**
			[# of accessed web] *Pages*	0.34
([13], p. 478)	Three years from 2007 to 2009.	Scholars on Bilbao (http://www.scholars-on-bilbao.info/)	*Number of visits*	0.68***

**Note:** Authors produced the Correlation Coefficients shown in the table.

* P-value ≤ 0.05; ** P-value ≤ 0.01; *** P-value ≤ 0.001 (student's *t*-test, two-tails, two equal and independent samples of variables).

According to the results of these four verification tests, the data of the ‘number of captures’ provided by Archive.org can be accepted as ‘web traffic’ data. Accordingly, all data records of CSR disclosure must be checked^7^, and the number of captures in each data record must be collected, as presented in Appendix 1. The unit of ‘web traffic’ shall be recognised as the Statistical Incidence of Stakeholders’ Usage of Online Data (SISUOD).

**Fig. 1:**
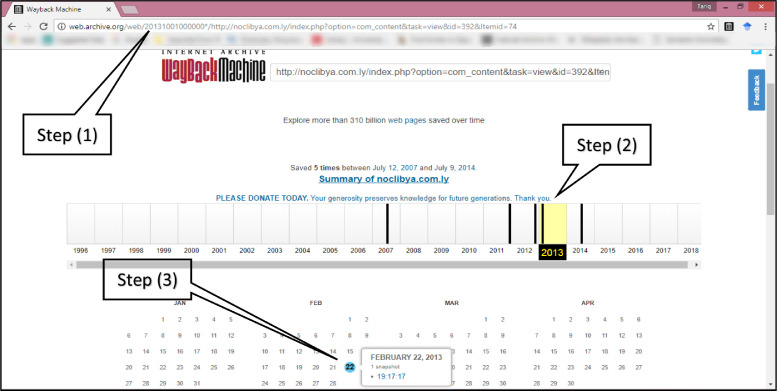
The required steps to collect 'number of snapshots' (web traffic) have been taken by Archive.org in specific webpage and specific year (this illustrated figure has been snapshotted on 06/01/2018).

The required steps to collect data, as illustrated in Fig. 1, start with displaying a report provided by Archive.org about the ‘summary of captures’ of the concerned web page throughout its lifetime. Fig. 1 shows the summary of captures of the selected web page^8^ prefixed by a specific URL^9^. This web page contains environmental information in the English language of 886 words. The second step is locating the year (period) to calendar-displayed details of all SISUODs in 2013. Each highlighted date contains the number of SISUODs on that date. The last step is moving the mouse pointer on that date to get an auto-displayed message with the number of SISUODs that should be recorded as web traffic, just one SISUOD. The last step (step 3) must be repeated as much as the highlighted dates found in the concerned year and, consequently, the number of SISUODs is the aggregation of snapshots in all highlighted dates. In this example, there is just one highlighted date, the 22nd of February 2013, as shown in Fig. 1. In fact, two SISUODs (the number of captures done by Archive.org) represent the volume of web traffic on this web page because the data records in this paper have been structured on a biennial basis, 2012–2013. Accordingly, I found just another SISUOD in the second year (2012) on the 2nd of November 2012. It is worth noting that the total number of SISUODs for this webpage is five, as shown in Fig. 1.

**Fig. 2:**
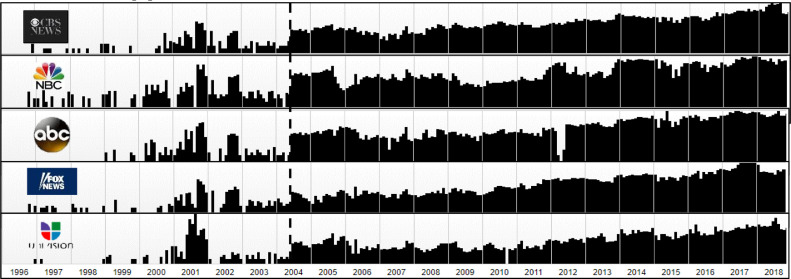
Five diagrams provided by www.archive.org plotted the number of times (small dark attached-bars) of capturing websites of the most popular channels in the world since this archive has been established in 1996.

This method of generating web traffic data can be used for any website (or web page) since Archive.org was established in 1996. However, I have noticed that the mechanism of snapshotting websites has become regular since the summer of 2004, as shown in Fig. 2. That moment of mature performance of Archive.org is indicated by the vertical dash line in Fig. 2. Before 2004, the SISOUDs of any website are unlikely to have been significantly associated with its real web traffic data. This is why I have collected SISOUDs every week using Monach University's Archive.org of 2005, as the real data of web traffic is for 2002.

## An empirical application

As mentioned before, the introduced method has been applied to CSR information published on the websites of 13 oil companies operating in Libya. The research time horizon covers eight years from 2008 to 2015. According to the collected data, not all web pages are launched exclusively for CSR information. The web traffic on this type of web pages is likely to be scored for various web contents rather than CSR information itself. Furthermore, there is no significant difference in the visual intentionality of displaying textual contents (e.g., coloured or flashing fonts, unique background colour) on any web page. All textual contents have been formatted similarly. Accordingly, the web traffic on CSR information (in each data record) should be adjusted by the difference between its textual size and the whole textual capacity of the web page (vehicle) at the date and time of publishing the CSR disclosure. I determined the (adjusted) web traffic of CSR information apart from non-CSR information, which both share the same web pages in different proportions. The final tabulation of the adjusted web traffic (the outputs of the introduced method) of all data records is presented in Table 2. It shows the estimated web traffic on five different types of CSR information found on the websites of oil companies of different types of ownerships.

**Table 2:** The web traffic (in SISOUDs) on CSR information of 13 major oil companies.

Website version	Types of disclosure	2008-2009				2010-2011				2012-2013				20,141–2015			
		JVCs	NCs	Owner	Total	JVCs	NCs	Owner	Total	JVCs	NCs	Owner	Total	JVCs	NCs	Owner	Total
Arabic	Community	2.1	11.8	45.8	59.7	26.1	9.9	74.9	110.9	15.6	16.0	33.2	64.8	1.7	12.3	6.1	20.1
	Customer		8.6		8.6		1.5	1.7	3.2		2.0	0.2	2.2	0.1	5.8	0.2	6.2
	Employee	9.2	45.0	56.5	110.7	38.9	46.5	38.7	124.1	3.4	20.4	53.1	76.9	1.0	18.1	6.9	26.0
	Environment	0.8	8.9	21.9	31.6	88.9	0.4	17.0	106.3	1.7	0.3	23.9	26.0	4.1	1.2	2.8	8.1
	HS&E	1.0	32.0	3.1	36.0	40.0	18.1	3.1	61.3	12.4	5.5		17.9	2.0	11.3		13.3
	Total	13.1	106.3	127.3	246.7	193.9	76.5	135.5	405.8	33.1	44.3	110.4	187.8	8.8	48.7	16.1	73.6
English	Community	2.0		13.1	15.1	4.2		11.1	15.3	1.4	1.8	1.1	4.3	61.4	7.9	3.9	73.2
	Customer	0.6		0.6	1.1						4.4		4.4	0.9	6.2	0.6	7.7
	Employee	65.6	0.0	0.5	66.1	1.9		0.4	2.3	1.0	21.6	1.7	24.3	0.0	31.6	0.6	32.3
	Environment	11.9	0.2	6.7	18.9	4.1		0.1	4.2	1.1	0.6	0.9	2.6	1.2	2.4	2.3	5.9
	HS&E	39.6	0.6		40.2	5.8	4.5	0.2	10.5	6.7	1.1		7.8	1.3	7.6	0.3	9.2
	Total	119.7	0.8	20.9	141.4	15.9	4.5	11.9	32.3	10.2	29.5	3.7	43.4	64.8	55.8	7.7	128.3
Eng. & Ara.	Community	4.1	11.8	58.9	74.8	30.3	9.9	86.0	126.2	17.0	17.8	34.3	69.1	63.1	20.2	10.0	93.3
	Customer	0.6	8.6	0.6	9.7		1.5	1.7	3.2		6.4	0.2	6.6	1.0	12.0	0.8	13.9
	Employee	74.8	45.0	57.0	176.8	40.8	46.5	39.1	126.4	4.4	42.0	54.8	101.2	1.0	49.7	7.5	58.3
	Environment	12.7	9.1	28.6	50.5	93.0	0.4	17.1	110.5	2.8	0.9	24.8	28.6	5.3	3.6	5.1	14.0
	HS&E	40.6	32.6	3.1	76.2	45.8	22.6	3.3	71.8	19.1	6.6		25.7	3.3	18.9	0.3	22.5
	Total	132.8	107.1	148.2	388.1	209.8	81.0	147.4	438.1	43.3	73.8	114.1	231.2	73.6	104.5	23.8	201.9

Indicates to non-disclosure figures and, consequently, no web traffic detected.

JVC Joint Venture Companies.

NCs National Companies.

Owner The holding company of all Libyan oil sector. It is called National Oil Corporation.

## Conclusion

The adopted approach in the research study could be considered as the embryonic stage of quantifying the concept of ‘stakeholders’ engagement’, especially on firms’ websites that are not supported by public ‘advocacy advertising’ [16] features to understand, qualitatively, stakeholders’ opinions and perceptions about online CSR contents.

This novel method has been established by re-defining statistical data at publicly available sources. It would be feasibly very helpful for researchers studying the stakeholders’ usage of web content in the CSR area or any other web-based research study. In other words, it could be the threshold of digitalizing stakeholders’ engagement with web-based content.

The adopted method in this paper of using web *metrics* to study stakeholders’ engagement and accessibility might guide firms to deepen their perceptions about stakeholders’ expectations and needs. Moreover, it provides them with updated indicators of stakeholders’ agreement about their expectations and needs. For example, the specification and prioritization of web-based CSR agendas can be longitudinally assessed using this method, mathematically determining the firm's awareness regarding the significance of web-based CSR hierarchy compared to the whole size of the website. A comprehensive picture of the institutional awareness of using such media should be drawn up by a dimensional analysis of the web-based CSR hierarchy, for example, how web CSR information is close to (or far from) the home page. This is applicable not only on the firms’ side as they are CSR communicators but also interprets the trends of stakeholders’ usage of web-based CSR content.

### Ethics statements

*MethodsX has ethical guidelines that all authors must comply with. In addition, we ask you to complete the relevant statement(s) below. Please delete those which are not relevant to your work.*

***If your work involved human subjects,*** *please include a statement here confirming that the relevant informed consent was obtained from those subjects:*

***If your work involved animal experiments,****please include a statement here confirming that those experiments complied with the* *ARRIVE guidelines* *and were carried out in accordance with the U.K. Animals (Scientific Procedures) Act, 1986 and associated guidelines;* *EU Directive 2010/63/EU for animal experiments**; or the National Institutes of Health guide for the care and use of laboratory animals (NIH Publications No. 8023, revised 1978). Note, the sex of the animals must be indicated, and, where appropriate, the influence (or association) of sex on the results of the study:*

***If your work involved data collected from social media platforms*** *, please include a statement here confirming that a) informed consent was obtained from participants or that participant data has been fully anonymized, and b) the platform(s)’ data redistribution policies were complied with:*

## CRediT authorship contribution statement

**Tariq S. Almontaser:** Methodology, Validation, Formal analysis.

## Declaration of Competing Interest

The authors declare that they have no known competing financial interests or personal relationships that could have appeared to influence the work reported in this paper.

## Data Availability

Data will be made available on request.

## Footnotes

1 http://europa.eu/rapid/press-release_EDPS-13-10_en.htm. Accessed on 12/03/2019.

2 Examples of paid services: Extreme Tracking http://www.extreme-dm.com, Web Site Traffic Report http://www.websitetrafficreport.com, Web Stat http://www.web-stat.com, Web Site Tracker http://www.websitetracker.com, Super Stats http://www.superstats.com and Free Stats http://www.freestats.com[15].

3 www.scholars-on-bilbao.info/.

4 https://archive.org/about/terms.php. Updated on 31st of December 2014. Accessed on 10/07/2016.

5 Number of captures is: the total number of hyperlinked and restorable backups, during a period of time, of a specific web page (or URL) have been chronologically crawled, and presented, by www.archive.org.

6 https://archive.org/about/faqs.php. Accessed on 01/07/2016.

7 http://web.archive.org/web/*/<URL of data record>

8 http://noclibya.com.ly/index.php?option=com_content&task=view&id=392&Itemid=74.

9 http://web.archive.org/web/*/.
